# Isolation and Characterization of bovine parainfluenza virus type 3 genotype C from brown cattle with respiratory disease in Yili, Xinjiang, China

**DOI:** 10.3389/fcimb.2026.1901710

**Published:** 2026-09-03

**Authors:** Xiuyu Zhang, Xingxing Liu, Guiling Wu, Qiang Fu, Lei zhang, Xintong Chen, Chuanfeng Wang, Jinxin Xie, Panpan Tong

**Affiliations:** 1 College of Veterinary Medicine, Xinjiang Agricultural University, Urumqi, China; 2 Aksu Regional Animal Disease Control and Diagnostic Center, Aksu, China; 3 Sino-science Gene Biological Technology Co., Ltd, Harbin, China; 4 Key laboratory for animal disease detection, College of Animal Sciences, Yili Vocational and Technical College, Yili, China; 5 Xinjiang Key Laboratory of New Drug Research and Development for Herbivores, Urumqi, China

**Keywords:** bovine parainfluenza virus type 3, brown cattle, China, genotype C, respiratory disease

## Abstract

**Purpose:**

In winter 2025, a severe respiratory disease outbreak occurred in brown cattle in Yili, Xinjiang, China.

**Results:**

We detected bovine parainfluenza virus type 3 (BPIV-3) in all clinical samples from affected animals and successfully isolated the virus. Whole-genome sequencing and phylogenetic analysis classified the isolates as genotype C, which is currently the predominant lineage circulating in Chinese cattle herds. Importantly, the key antigenic epitopes of the HN and F proteins were fully conserved compared with the approved vaccine strain SD0835.

**Conclusion:**

This study provides the first molecular evidence of BPIV-3 genotype C as the causative agent of a respiratory disease outbreak in brown cattle in Xinjiang, and the epitope conservation suggests that the existing vaccine may offer cross-protective efficacy. Our findings underscore the need for enhanced surveillance and prompt implementation of preventive strategies in this region.

## 1 Introduction

Bovine respiratory disease complex (BRDC) causes significant economic losses to the livestock industry. The pathogens that induce BRDC are diverse, mainly including Mycoplasma bovis (M. bovis), Pasteurella multocida (Pm), Mannheimia haemolytica (Mh), infectious bovine rhinotracheitis virus (IBRV), bovine viral diarrhea virus (BVDV), bovine respiratory syncytial virus (BRSV), bovine coronavirus (BCoV), and bovine parainfluenza virus type 3 (BPIV-3) (Chai et al., 2022; Kamel et al., 2024; Ban et al., 2026; Zhang et al., 2025). BPIV-3 belongs to the genus Respirovirus within the family Paramyxoviridae. It is an enveloped, non-segmented virus containing a negative-sense single-stranded RNA genome of approximately 15.5 kb. The viral genome encodes six structural proteins (nucleocapsid, phosphoprotein, matrix, F, HN, L) and three non-structural ones (C, V, D) (Xu et al., 2024). BPIV-3 is recognized as one of the major viral pathogens involved in bovine respiratory disease complex (BRDC). BRDC is a multifactorial disease characterized by mixed infections, leading to high morbidity, reduced calf survival, and impaired production performance in adult cattle (Miles, 2009; Ellis, 2010; Zhu et al., 2011). Phylogenetic analyses have classified BPIV-3 into three major genotypes, designated A, B, and C (Zhu et al., 2011; Ren et al., 2023). Infection with BPIV-3 can induce respiratory epithelial injury and immunosuppression, thus predisposing affected animals to secondary bacterial or mycoplasma infections that further aggravate pneumonia and mortality (Werid et al., 2026).

In China, intensive cattle farming and animal transport boost BPIV-3 detection. Rates exceed 50% in some regions (Ren et al., 2023). Despite the widespread circulation of the virus, commercially available vaccines and precise diagnostic tools remain limited. Xinjiang is one of the major cattle-producing regions in China and is characterized by extensive natural pastures and the continued expansion of large-scale dairy and beef farming systems. In northern Xinjiang (Yili, Tacheng, Altay), long cold winters force crowded, poorly ventilated indoor housing, promoting BRDC. Recent severe calf respiratory disease (cough, nasal discharge, dyspnea, mortality) on northern Xinjiang large farms suggests suspected BRDC outbreaks (Xu et al., 2024; Ren et al., 2023). However, compared with studies conducted in central and northern China, systematic investigations regarding the prevalence, dominant genotypes, genetic evolution, and pathogenic characteristics of BPIV-3 in Xinjiang remain limited. This lack of regional epidemiological data has restricted the development of effective prevention and control strategies for BRDC in this area.

In the winter of 2025, a large-scale respiratory disease outbreak occurred among brown cattle in Yili, Northern Xinjiang, China. Affected animals displayed typical respiratory symptoms, including cough, increased secretions, dyspnea, fever (> 39.5 °C). Mortality was primarily observed in calves younger than 5 months of age, with a case fatality rate of 30%. The aim is to identify the causative agent of a severe respiratory disease outbreak in brown cattle in Yili, Xinjiang, and characterize the circulating strain of BPIV-3 at molecular and phylogenetic levels to understand its epidemiological role.

## 2 Materials and methods

### 2.1 Sample collection

**Figure 1:**
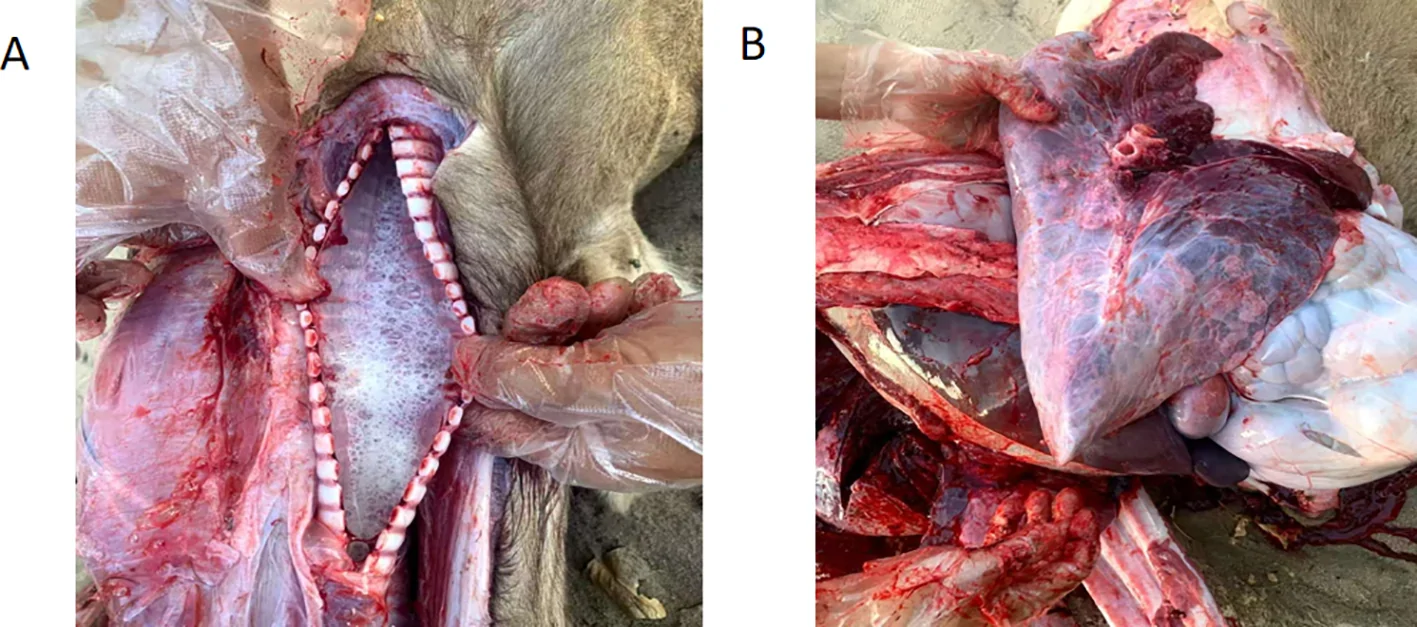
Lung and trachea of calves that died from respiratory diseases. **(A)** Trachea. **(B)** Lung.

In the winter of 2025, a respiratory disease outbreak occurred in a brown cattle herd in Yili. The local veterinarians submitted 238 samples, including 180 nasal swabs, and 48 lung tissue samples (**Figure 1**) collected from diseased and dead calves, as well as 10 nasal swab samples from healthy cattle. Nasal swabs were placed into test tubes containing 10 mL phosphate buffer solution. Lung tissues were collected in sterile bags and stored at −80 °C until further analysis.

### 2.2 Viral metagenomic analysis

For meta-transcriptomics (MTT), an RNA library was constructed Novogene Co., Ltd. Briefly, the 10 samples were pooled, homogenized, and 400 μL supernatant was concentrated by ultracentrifugation was treated with 1,200 μL Trizol reagent (Tiangen, China), and total RNA was extracted according to the manufacturer’s instructions. Ribosomal RNA (rRNA) was then removed using the Epicenter Ribo-zero rRNA Removal Kit (Epicenter, USA), followed by ethanol precipitation to eliminate residual rRNA-free components. Sequencing libraries were then prepared from the rRNA-depleted RNA using the Fast RNA-seq Lib Prep Kit V2 (Abclonal, USA, Cat. No. RK20306) according to the manufacturer’s protocol (Li et al., 2025).

For the establishment of the DNA library by multiple displacement amplification (MDA), DNA was extracted from the filtered supernatant using the DNeasy Blood & Tissue Kit (QIAGEN GmbH, Germany). The sequencing library was then prepared using the GenomiPhi V2 DNA Amplification Kit (Cytiva, UK). DNA concentrations were measured using the Qubit 1× dsDNA HS Assay Kit (Invitrogen, USA). The constructed RNA and DNA libraries were sequenced on the Illumina NovaSeq 6000 platform (Novogene, Tianjin, China), generating approximately 6G of data for each library, which provided sufficient sequencing depth for viral screening (Li et al., 2025).

Cleaned data generated by HTS were initially aligned against the cattle genome sequence using BWA 0.7.17. Unaligned reads were extracted using Samtools 1.17. The filtered sequence data were then *de novo* assembled into contigs using Megahit 1.2.9 based on the Kmer iterative DBG approach. The assembled contigs were further aligned and annotated against the nr and nt databases of NCBI using Diamond 2.1.8 and Nucleotide–Nucleotide BLAST 2.13.0 +. Annotation results were further verified online using the Blastx module of the NCBI database. After screening and removal of nonviral genetic sequences, the confirmed viral sequences were aligned with the assembled contigs, and the number of viral reads was quantified using Bowtie2 version 2.4.5 (Li et al., 2025).

### 2.3 Respiratory pathogen detection

Each nasal swab sample was vortexed, and each lung tissue sample was homogenized and centrifuged. A 200 μL aliquot of the supernatant was then collected from each sample. Following the manufacturer’s instructions, viral RNA/DNA was extracted using a kit from Geneaid Biotech Co (Cat. No. VR100)., and bacterial or mycoplasma DNA was extracted using a kit from Tiangen Biotech (Beijing) Co., according to the manufacturer’s instructions. Reverse transcription was performed using Reverse Transcriptase M-MLV (Takara Biotech Co.). To confirm the presence of *M. bovis*, Pm, Mh, IBRV, BVDV, BRSV, BCoV, and BPIV-3, PCR was performed using the primers listed in **Table 1** (Zhang et al., 2025), and amplification was carried out with 2×UltraHiFi Mix II (Tiangen Biotech Co.). The thermocycling conditions were as follows: initial denaturation at 98 °C for 1 min; 35 cycles of 98 °C for 10 s, 60 °C for 30 s, and 72 °C for 10 s; followed by a final extension at 72 °C for 5 min.

**Table 1:** Primer sequences of BPIV.

Primer name	Primer sequence (5’-3’)	Product length (bp)	References
Mb-F	CGTTATGCAAGATTAAATACTTACGAC	450	Zhang et al., 2025
Mb-R	TGAAAACTTTCTCAGCATTAGCC		
Mh-F	GCAGGAGGTGATTATTAAAGTGG	206	
Mh-R	CAGCAGTTATTGTCATACCTGAAC		
Pm-F	ATCCGCTATTTACCCAGTGG	460	
Pm-R	GCTGTAAACGAACTCGCCAC		
IBRV-F	TCTCGACCGGGGACATTATC	365	
IBRV-R	GCCTCTTCGATCACGCAGTC		
BVDV-F	GCCATGCCCTTAGTAGGACTAGC	290	
BVDV-R	CAACTCCATGTGCCATGTACAGC		
BRSV-F	TATGCTATGTCCCGATTGG	569	
BRSV-R	ACTGATTTGGCTAGTACACCC		
BCoV-F	AAGGTGTGCCTATTGCACCAG	500	
BCoV-R	GCTTAGTTACTTGCTGTGGC		
BPIV-3-F	AGTGATCTAGATGAWGATCC	329	
BPIV-3-R	GTTAYTGAKCCAATWGCTGT		

### 2.4 Isolation, screening, indirect immunofluorescence assay (IFA), and electron microscopy of BPIV-3

*In vitro* isolation of BPIV-3 was performed using MDBK cells, as previously described (Xu et al., 2024). Supernatants from 34 randomly selected BPIV-3-positive samples were filtered through a 0.22 μm membrane filter, and the filtrates were inoculated onto MDBK cells. The inoculated cells were incubated at 37 °C in a 5% CO_2_ incubator for 2 h. After adsorption, the inoculum was discarded and replaced with DMEM supplemented with 2% fetal bovine serum (FBS). The cells were maintained for 72 h, followed by three freeze–thaw cycles for virus harvesting. The harvested viruses were then passaged continuously for six generations in MDBK cells. Cytopathic effect (CPE) was monitored daily after inoculation, and amplification of the BPIV-3 L gene was performed to confirm BPIV3 infection as the initial respiratory pathogen detection assay. For IFA, MDBK cells were seeded into 6 wells of a 12-well culture plate and allowed to grow until they reached 70-80% confluency. Four wells were inoculated with the previously prepared virus solution, while the remaining two were negative controls. The cells were then incubated for 24 hours. Following incubation, the cells were fixed with 4% formaldehyde for 30 minutes, then blocked with 1% bovine serum albumin (BSA) at room temperature for 30 minutes. A mouse polyclonal antibody targeting the BPIV3 (kindly provided by Prof. Xin Yin) was applied at a 1:5000 dilution, and the plates were incubated for 1 hour at 37 °C. After washing with phosphate-buffered saline containing Tween 20 (PBST), the cells were incubated with Alexa Fluor 488-conjugated donkey anti-mouse IgG (Invitrogen) at a 1:1000 dilution for 2 hours at 37 °C. Following a final wash with PBST, fluorescence was visualized using an Zeiss LSM 980 confocal laser scanning microscope at 10× magnification. For electron microscopy, BPIV-3 particles were negatively stained with 2% phosphotungstic acid before observation.

### 2.5 Viral whole genome sequencing (WGS)

The whole-genome sequences of two BPIV-3 were obtained using meta-transcriptomics (MTT). Briefly, 400 μL of each isolate was treated with 1,200 μL Trizol reagent (Tiangen, China), followed by the construction of two RNA libraries, high-throughput sequencing, and bioinformatic analysis as described above for viral metagenomic analysis (Li et al., 2025).

### 2.6 Multiple sequence alignment and phylogeny

**Figure 2:**
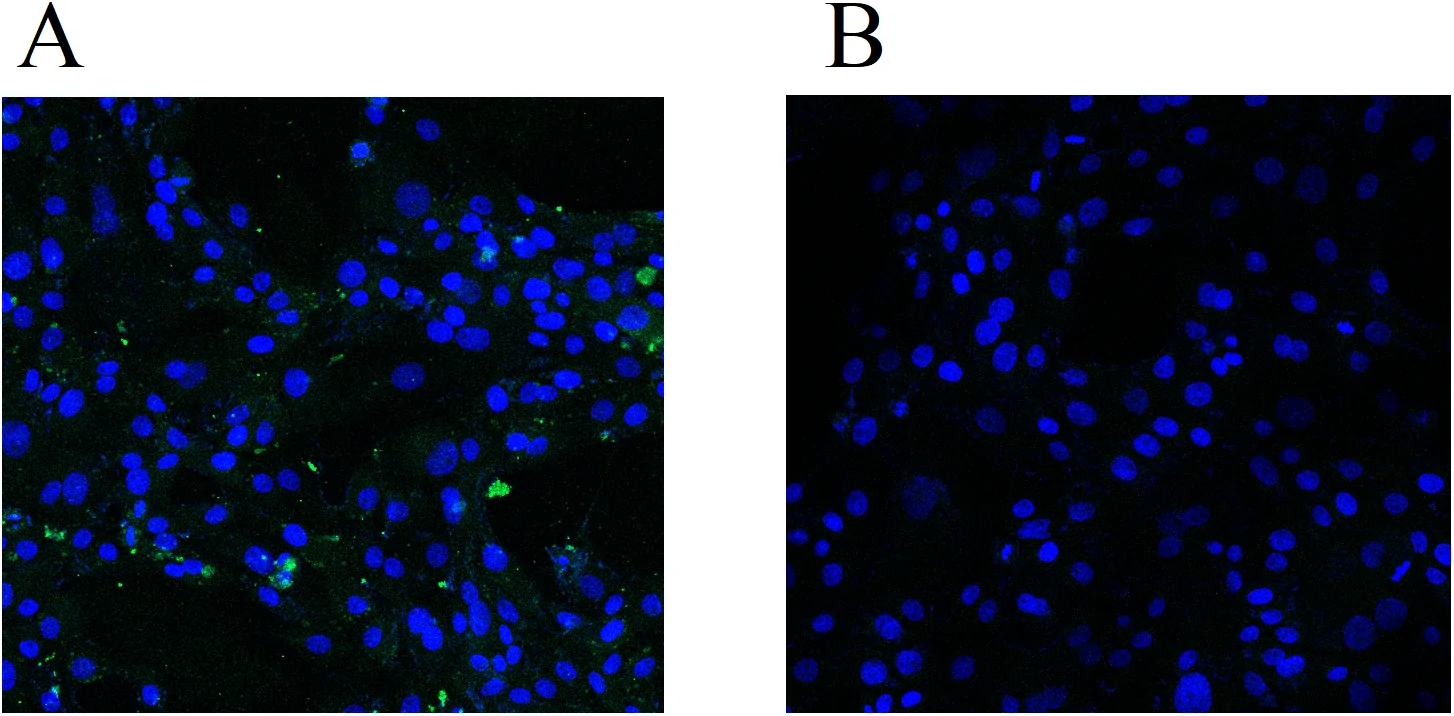
IFA for BPIV-3. **(A)** IFA detection of BPIV-3-infected MDBK cells using an anti-BPIV-3 monoclonal antibody. **(B)** MDBK cells negative for BPIV-3 were used as the control.

The GenBank accession numbers of the sequences included in this study are presented in **Figure 2**. Two whole-genome sequences were deposited in GenBank under accession IDs PZ015910 and PZ015911. Multiple sequence alignment was performed using MegAlign (Lasergene v7.1). Phylogenetic analysis was conducted using MEGA7, and a maximum-likelihood phylogenetic tree was constructed based on the Tamura–Nei model. The robustness of the phylogenetic tree topology was assessed using 1,000 bootstrap replicates (Kumar et al., 2016).

## 3 Result

### 3.1 Overview of the virome

Metagenomic analysis of 10 lung samples collected from dead calves showing respiratory disease, yielded 28,801,700 sequencing reads. Among these, 1,397,980 reads (4.9%) were annotated as mammalian viral sequences, including BPIV-3, BVDV, and retroviruses. Of the mammalian viral reads, 1,375,911 were 150 nucleotides (nt) in length and displayed 99.6 – 99.7% nt identity with various genes of BPIV-3 reference strain XJ20255-3 (GenBank accession no. OM632676).

### 3.2 Isolation and identification of BPIV-3

PCR analysis confirmed BPIV-3 in all 228 nasal swab and lung tissue samples, BVDV in seven lung tissue samples, and *M. bovis* in 12 nasal swabs and four lung tissue samples collected from diseased and deceased calves. However, these pathogens were not detected in nasal swab samples from 10 healthy cattle. These findings indicate BPIV-3 as a potential causative agent associated with the respiratory disease outbreak among brown cattle in Yili, Xinjiang, China.

**Figure 3:**
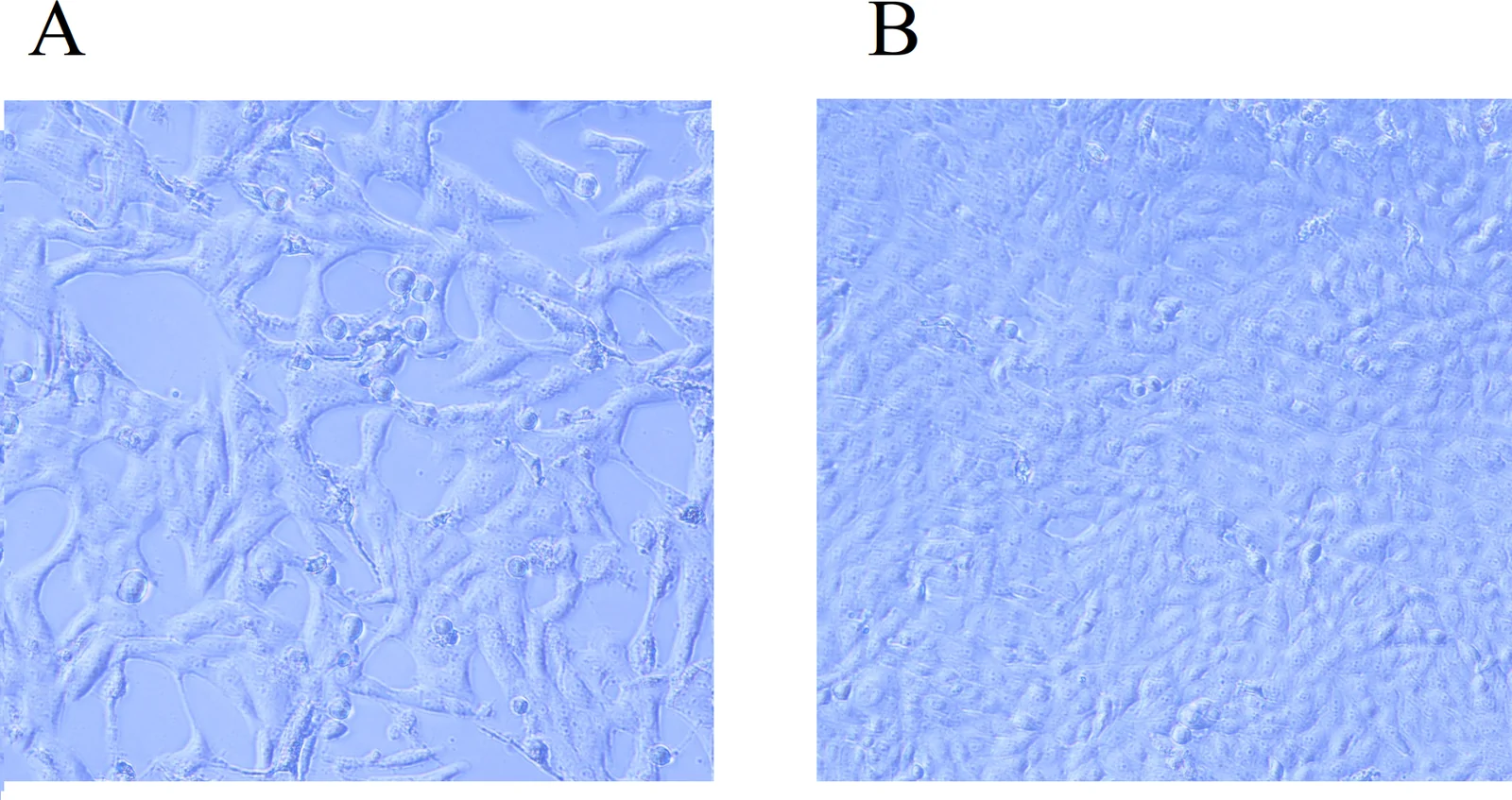
Cell morphology following inoculation (×100). **(A)** CPEs observed in infected cells. **(B)** Normal control cells.

**Figure 4:**
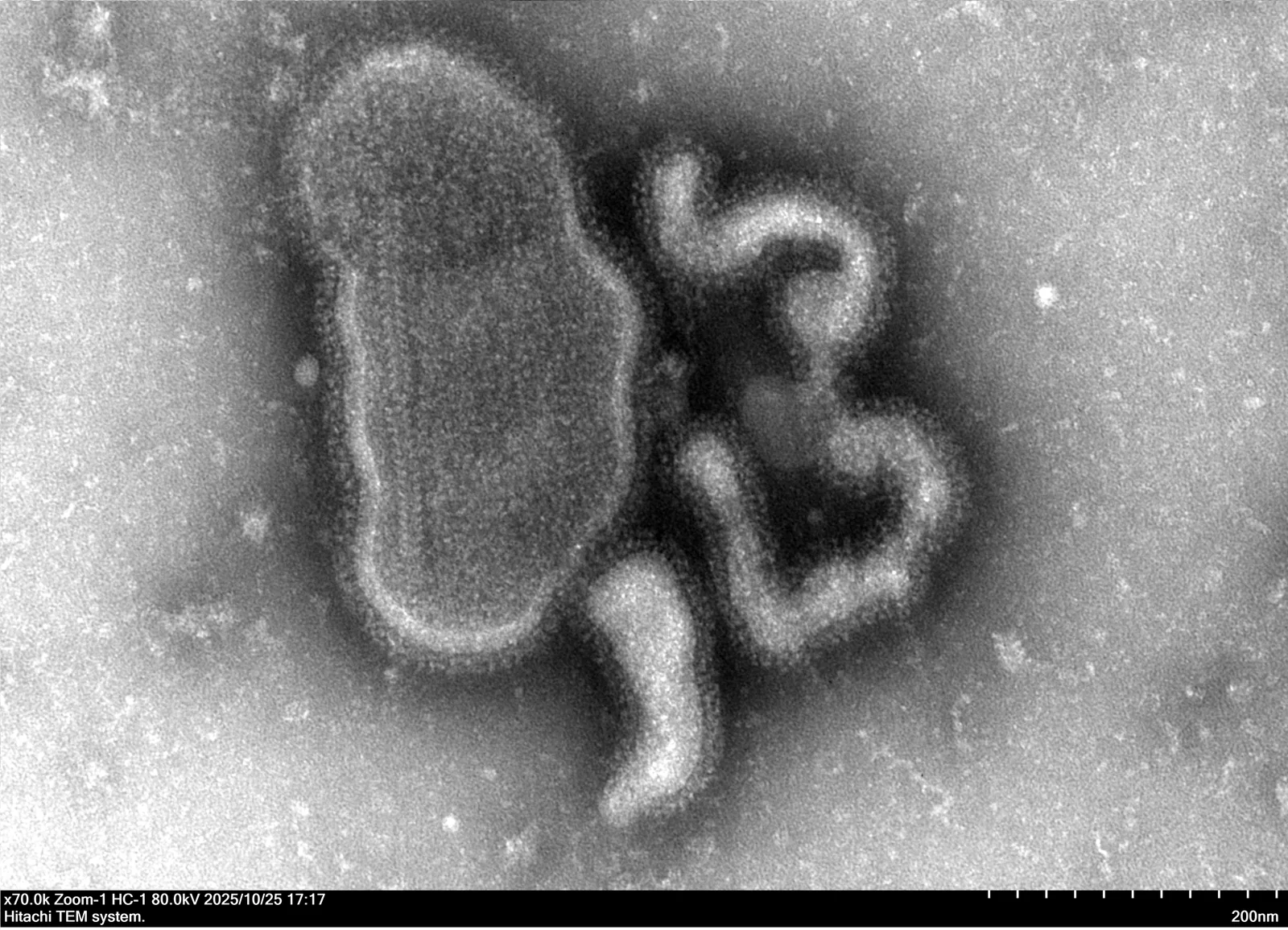
TEM image of BPIV-3 particles (Hitachi HT7800, Japan).

Thirty-four of the 228 BPIV-3-positive samples were randomly selected for virus isolation. Cytopathic effects (CPE) were observed in a few isolates at passage one, whereas most isolates developed CPE by passage four; all isolates showed clear CPE by the sixth passage in MDBK cells. Infected MDBK cells showed characteristic morphological changes, including cell rounding, necrosis, and formation of reticular structures with cytoplasmic vacuoles at 24 h post-infection, compared with non-infected control cells (**Figure 3**). The presence of BPIV-3 further confirmed by RT-PCR, IFA (**Figures 2A, B**), and its identity was validated via transmission electron microscopy (TEM), which revealed viral particles with typical morphological features of BPIV-3, with an approximate virion diameter of ~250 nm (**Figure 4**). Virus isolates were designated XJ2024, XJ2025, XJ20251 to XJ202532.

### 3.3 Whole-genome sequence evaluations and phylogenetic inferences

**Figure 5:**
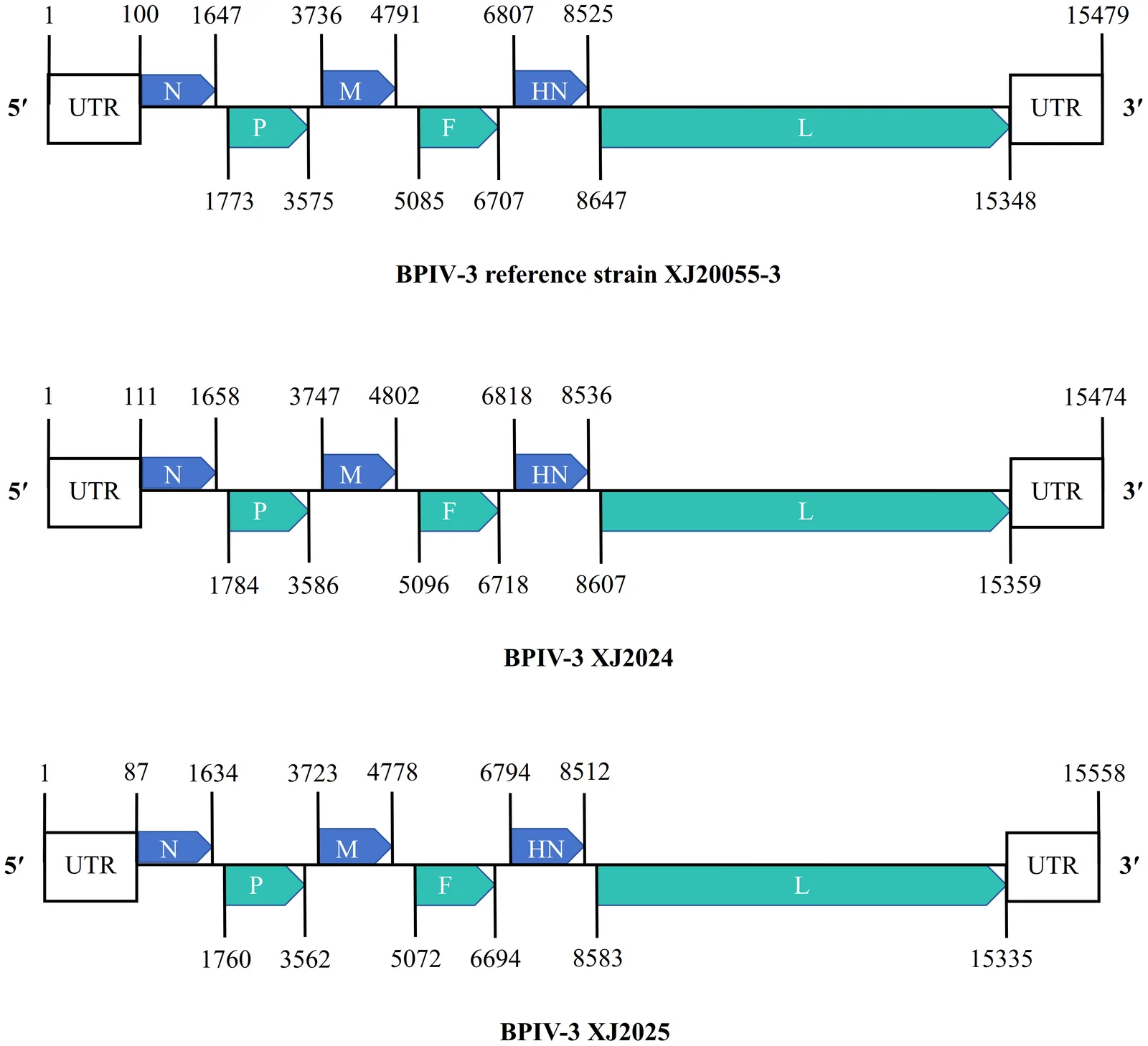
The genome structure of the BPIV-3 XJ-2024, and XJ-2025 (GenBank accession numbers PZ015910 and PZ015911) identified in brown cattle from China.

To investigate the molecular characteristics of the isolates, we performed viral metagenomic sequencing on two randomly selected BPIV-3 isolates, XJ2024 and XJ2025. The two runs generated total reads of 91,610,358 and 108,695,908, respectively, of which 1,172,581 (2.8%) and 1,413,721 (1.3%) reads were annotated as BPIV-3. These reads were assembled into two complete genomes, with lengths of 15,474 nt and 15,558 nt, respectively. Genome organization analysis revealed that the genomic structures of the isolates are consistent with that of the reference strain XJ20055-3 (**Figure 5**). Comparative genomic analysis showed that XJ2024 and XJ2025 shared 100% nucleotide identity. Both isolates displayed 98.0 – 99.7% identity with BPIV-3 genotype C reference strains XJ20255-3, NMTL-21, TVMDL16, 87043-2004, and etc (**Table 2**), whereas they showed less than 82.3% identity with genotype A and B reference strains NM09, BJ, TVMDL15, and XJ21032-1, confirming their classification within BPIV-3 genotype C (**Table 2**).

**Table 2:** Homology analysis of the full genome of BPIV3 XJ2024 and XJ2025 in this study.

Genotype	Isolate	Nucleotides (%)
C	BPIV3 XJ20055-3 BPIV3 NMTL-21 BPIV3 SC20 BPIV3 LT2 BPIV3 HB2 BPIV3 NM2 BPIV3 NX49 BPIV3 SC39 BPIV3 NX4 BPIV3 SX2021 BPIV-3C/Bovine/CHN/Hubei-01/2021 BPIV3 JL6 BPIV3 SD0835 BPIV3 XJKS-2023 BPIV3 Sweden/SLU-K-1/2016 BPIV3 87043-2004 BPIV3 TVMDL16	99.7 99.7 99.7 99.7 99.7 99.7 99.7 99.6-99.7 99.6 99.6 99.5 99.3-99.4 99.3 99.1 98.5 98.2 98.0
A	BPIV3 NM09 BPIV3 BJ	82.3 82.1
B	BPIV3 TVMDL15 BPIV3 XJ21032-1	81.4-81.5 81.0

**Figure 6:**
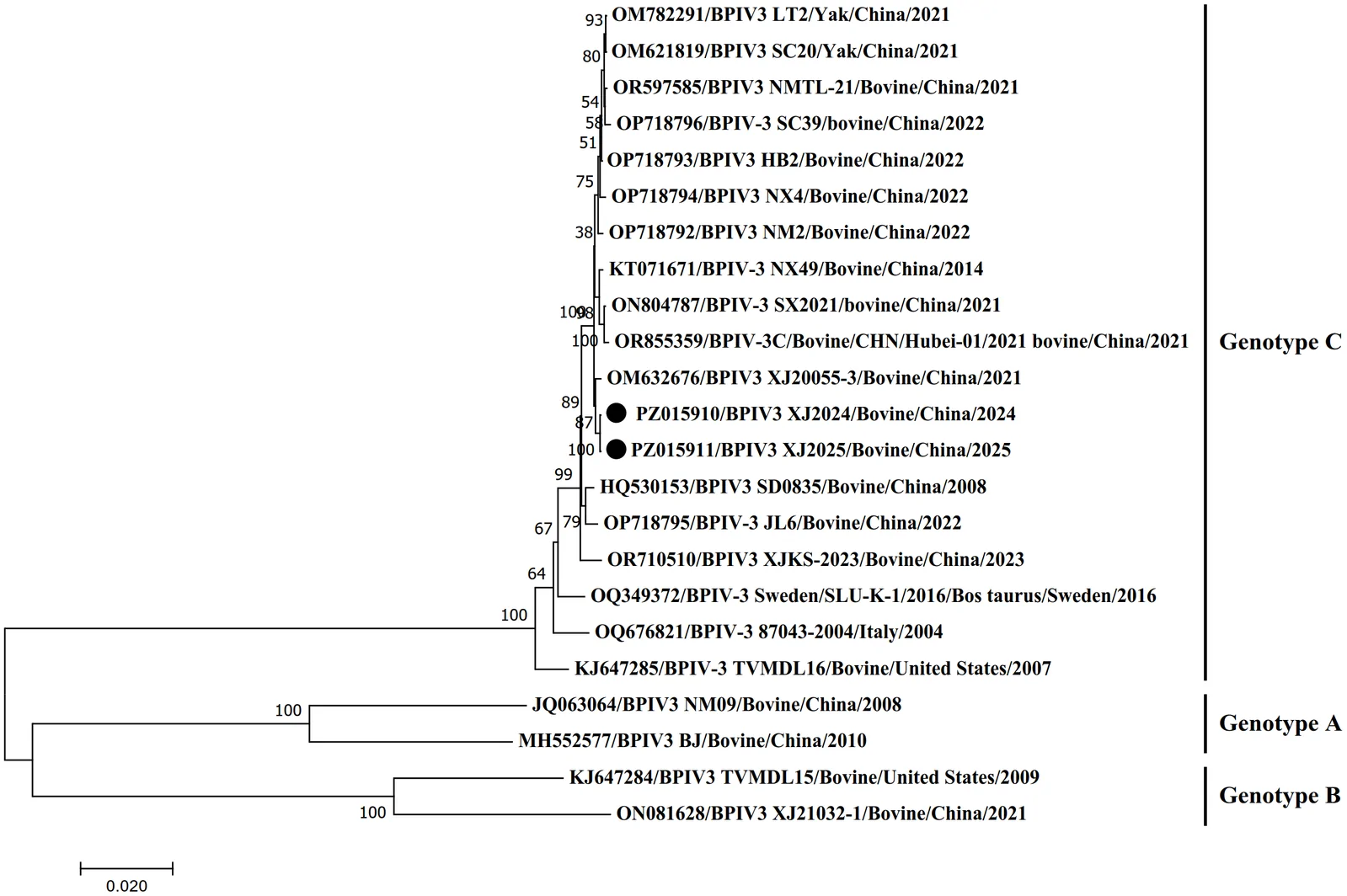
Phylogenetic analysis of XJ2024 and XJ2025 isolates. WGS of the two isolates, together with reference strains, were used to construct a maximum-likelihood phylogenetic tree in MEGA7 with 1,000 bootstrap replicates. Solid circles indicate BPIV-3 XJ2024 and XJ2025 isolates\identified in this study.

Phylogenetic analysis based on whole-genome sequences further demonstrated that XJ2024 and XJ2025 clustered within the BPIV-3 genotype C lineage. The isolates were closely related to established genotype C reference strains, including XJ20255-3, NMTL-21,TVMDL16, 87043-2004, and etc (**Figure 6**). The isolates grouped within the same evolutionary branch as XJ20255-3, NMTL-21, SC20, LT2, HB2, NX4, and NM2, while XJKS-2023 and the recently approved vaccine strain SD0835 in China formed a distinct branch. The clustering pattern further supports that the isolates belong to BPIV-3 genotype C and share close genetic relatedness with strains circulating within this lineage (**Figure 6**).

### 3.4 Amino acid mutation analysis of HN and F proteins of the isolates

**Figure 7:**
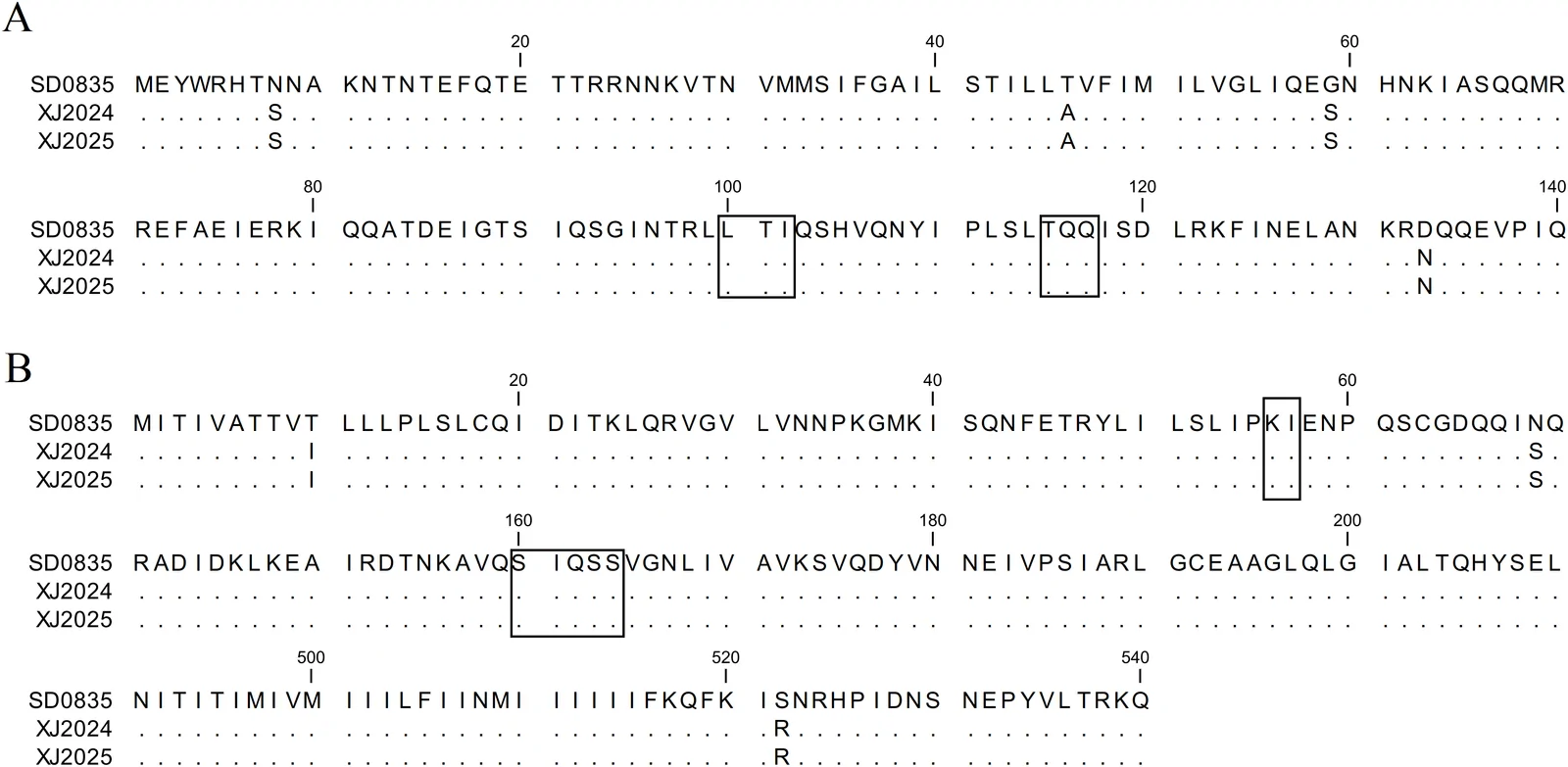
Amino acid sequence analysis of HN and F proteins. **(A)** Amino acid variation in the HN protein of the isolates. **(B)** Amino acid variation in the F protein of the isolates. Dots represent conserved residues relative to the approved vaccine strain BPIV-3 genotype C SD0835 (CLC Sequence Viewer 8).

Amino acid substitutions in the HN and F proteins of the isolates were analyzed in comparison with the approved vaccine strain SD0835. In the HN protein, mutations were identified at positions 8 (Serine, S), 46 (Alanine, A), 59 (Serine, S), and 133 (Asparagine, N). In the F protein, amino acid substitutions were observed at positions 10 (Isoleucine, I), 69 (Serine, S), and 522 (Arginine, R). No variations were detected in the reported antigenic epitope regions of the HN protein at positions 100–102 (Leucine–Threonine–Isoleucine, LTI) and 115–117 (Threonine–Glutamine–Glutamine, TQQ), nor in the F protein at positions 56–57 (Lysine–Isoleucine, KI) and 160–164 (Serine–Isoleucine–Glutamine–Serine–Serine, SIQSS) between the isolates and the vaccine strain (**Figure 7**).

## 4 Discussion

BPIV-3 represents a major threat to the global cattle industry, particularly in cattle herds in China (Chai et al., 2022; Kamel et al., 2024; Ban et al., 2026; Zhang et al., 2025). It is one of the core pathogens associated with BRDC, commonly known as “shipping fever” (Xu et al., 2024). The pathogenic impact of this virus is not limited to its direct effects; it also contributes to immunosuppression, increasing susceptibility to secondary infections and resulting in significant economic losses (Xu et al., 2024; Zhao, 2023). The impact of BPIV-3 on cattle production systems is systemic. Infection leads to reduced feed intake, impaired weight gain, and decreased milk yield, thus lowering overall production efficiency. Under stress conditions such as transport, weaning, or abrupt weather changes, outbreaks of BRDC become more severe and widespread, often causing high morbidity and mortality. Reported morbidity in feedlot populations can reach 60 – 90% (Zhao, 2023). Diagnosis, treatment, quarantine, and culling for outbreaks greatly increase production costs.

In China, the burden of BPIV-3 is particularly significant (Yi et al., 2023; Wang et al., 2022; Zhu et al., 2011; Bi et al., 2026; Werid et al., 2024; Zhou et al., 2025). As the world’s third-largest cattle-producing country, China’s herds face considerable epidemiological pressure. Surveillance studies indicate that more than 87% of cattle populations are affected by BRDC, with BPIV-3 identified as one of the major contributing pathogens. A multi-provincial investigation encompassing 16 provinces reported that BPIV-3 positivity among samples collected from farms experiencing BRDC outbreaks reached 18.17% (Ren et al., 2023). In 2016, a genotype C strain was isolated from a cattle farm in Xinjiang. Subsequent studies further demonstrated a detection rate of 4.4% in Angus breeding farms in southern Xinjiang, confirming ongoing viral circulation and highlighting its significant threat to the beef cattle industry in the region (Yi et al., 2023).

In the winter of 2025, following seasonal migration (turnout), a severe respiratory disease outbreak was reported in a herd of brown cattle in Yili, Xinjiang, China. The case fatality rate was around 7% in cattle older than 5 months, while it increased up to 30% in calves younger than 5 months. Bovine respiratory pathogens included the following: BPIV-3, BVDV, *M. bovis*, Pm, Mh, IBRV, BRSV, and BCoV (Chai et al., 2022; Kamel et al., 2024; Ban et al., 2026; Zhang et al., 2025; Mehinagic et al., 2019). In this study, viromics only annotated the bovine respiratory viruses BPIV-3 and BVDV. RT-PCR analysis detected BPIV-3 in all samples collected from diseased and deceased calves, whereas healthy calves were virus-negative. Additionally, seven of 48 lung tissue samples were positive for BVDV, and 12 of 180 nasal swabs from calves with respiratory disease, as well as four of 48 lung tissue samples, were positive for *M. bovis*. Despite the presence of these other pathogens and the limitations of the healthy control group, these results provide strong evidence implicating BPIV-3 as the causative agent.

Previous studies have classified BPIV-3 strains into three genotypes: A, B, and C (Xu et al., 2024; Ren et al., 2023). In this study, molecular characterization and phylogenetic analysis revealed that the isolated BPIV-3 strains belong to genotype C, which is consistent with recent epidemiological findings from multiple regions in China. For instance, Isolates from several Chinese provinces are predominantly genotype C (Ren et al., 2023; Yi et al., 2023; Wang et al., 2022; Zhu et al., 2011; Bi et al., 2026; Zhou et al., 2025). This confirms its dominance in cattle. Notably, the two isolates obtained in this study share 98.0%–99.7% whole-genome nucleotide identity with the earlier Xinjiang isolate XJ20255-3 (2016), indicating that BPIV-3 genotype C has been persistently circulating in Xinjiang for nearly a decade with relatively slow genetic evolution. This high level of genetic conservation suggests that no strong selective pressure has driven lineage replacement.

Previous studies have demonstrated that both the HN and F proteins of BPIV-3 serve as major protective antigens. The reported antigenic epitopes of the HN protein are located at amino acid positions 100–102 (LTI) and 115–117 (TQQ), whereas those of the F protein are mapped to positions 56–57 (KI) and 160–164 (SIQSS) (Coelingh et al., 1986). Although the epidemic strain identified in this study contains several amino acid substitutions across the overall protein sequences when compared with the approved vaccine strain SD0835, the amino acid composition within these key epitope regions remains fully conserved. This conservation suggests that vaccine-induced immune responses are theoretically capable of recognizing currently circulating BPIV-3 strains, indicating strong cross-reactive antigenic potential. Therefore, the approved vaccine may have the capacity to limit transmission of BPIV-3-associated respiratory disease in Xinjiang brown cattle populations. However, this assumption requires further experimental validation and field-based evaluation for confirmation of its protective efficacy under clinical conditions.

This outbreak caused significant economic losses to the brown cattle industry in Yili, which can be attributed to several interrelated factors. First, vaccination is considered the most effective strategy for preventing BPIV-3 infection. Although a BPIV-3 vaccine has been approved in China, it has not yet been commercialized, limiting its field application and preventive coverage. Second, BPIV-3 infection was followed by secondary infection with *M. bovis*, leading to the development of BRDC and resulting in a mortality rate as high as 30% in calves younger than 5 months. This finding is consistent with previous reports (Zhang et al., 2025; Mehinagic et al., 2019). Third, due to treatment costs exceeding 200 RMB, some herders delayed or provided inadequate therapeutic interventions, thereby accelerating disease progression and missing opportunities for effective outbreak control.

In conclusion, this study identifies BPIV-3 genotype C as the primary etiological agent associated with the severe respiratory disease outbreak in Yili, Xinjiang, during winter 2025. High detection rates, successful virus isolation, and whole-genome sequencing findings support active circulation of this genotype within the affected herd. The absence of a commercially available BPIV-3 vaccine in China remains a major limitation for effective disease prevention. Repeated detection of genotype C in Xinjiang and other regions suggests persistent circulation of this lineage and highlights the importance of prioritizing genotype C in future vaccine development strategies. Strengthening biosecurity measures, improving outbreak management, and implementing timely therapeutic intervention may help reduce BPIV-3-associated morbidity, mortality, and economic losses in cattle populations.

## Data Availability

The data presented in this study was deposited in the GenBank repository, accession numbers: PZ015910 and PZ015911.
